# *Mycoplasma bovis* NADH oxidase functions as both a NADH oxidizing and O_2_ reducing enzyme and an adhesin

**DOI:** 10.1038/s41598-017-00121-y

**Published:** 2017-03-03

**Authors:** Gang Zhao, Hui Zhang, Xi Chen, Xifang Zhu, Yusi Guo, Chenfei He, Farhan Anwar Khan, Yingyu Chen, Changmin Hu, Huanchun Chen, Aizhen Guo

**Affiliations:** 1 National Key Laboratory of Agricultural Microbiology, Huazhong Agriculture University, Wuhan, 430070 China; 2 College of Veterinary Medicine, Huazhong Agricultural University, Wuhan, 430070 China; 3 Key Laboratory of Development of Veterinary Diagnostic Products, Ministry of Agriculture, Huazhong Agricultural University, Wuhan, 430070 China; 4 Hubei International Scientific and Technological Cooperation Base of Veterinary Epidemiology, Huazhong Agricultural University, Wuhan, 430070 China

## Abstract

*Mycoplasma bovis* causes considerable economic losses in the cattle industry worldwide. In mycoplasmal infections, adhesion to the host cell is of the utmost importance. In this study, the amino acid sequence of NOX was predicted to have enzymatic domains. The *nox* gene was then cloned and expressed in *Escherichia coli*. The enzymatic activity of recombinant NOX (rNOX) was confirmed based on its capacity to oxidize NADH to NAD^+^ and reduce O_2_ to H_2_O_2_. The adherence of rNOX to embryonic bovine lung (EBL) cells was confirmed with confocal laser scanning microscopy, enzyme-linked immunosorbent assay, and flow cytometry. Both preblocking EBL cells with purified rNOX and preneutralizing *M. bovis* with polyclonal antiserum to rNOX significantly reduced the adherence of *M. bovis* to EBL cells. *Mycoplasma bovis* ^NOX–^expressed a truncated NOX protein at a level 10-fold less than that of the wild type. The capacities of *M. bovis* ^NOX–^ for cell adhesion and H_2_O_2_ production were also significantly reduced. The rNOX was further used to pan phage displaying lung cDNA library and fibronectin was determined to be potential ligand. In conclusion, *M. bovis* NOX functions as both an active NADH oxidase and adhesin, and is therefore a potential virulence factor.

## Introduction

*Mycoplasma bovis* is a major bacterial pathogen, causing pneumonia, mastitis, and arthritis in cattle throughout the world^1–4^. It was first identified in milk from cows with mastitis in 1961 and was reported to be responsible for pneumonia in the USA in 1976^2^. In China, *M. bovis* was first isolated from lung lesions in a calf with pneumonia in 2008. Although *M. bovis* has been known for 55 years, little progress has been made in clarifying the mechanism of its pathogenesis. However, the complete genome sequences of *M. bovis* type strain PG45^5^ and Chinese strains Hubei-1^6^ and HB0801^7^ have provided useful information for identifying the virulence factors of *M. bovis*. As is well known, adhesion to the host cell is an important and first step in successful bacterial infection. Therefore, adhesins are essential for bacterial pathogenesis. Fimbriae or cell wall components are the usual candidate adhesion molecules. However, because the dramatic genomic downsizing of the mycoplasmas has resulted in the loss of the cell wall, the membrane and membrane-associated proteins are believed to play significant roles in mycoplasmal adhesion^8^. In *M. bovis*, several membrane proteins, including lipoprotein P26^9^, a VpmaX-like lipoprotein^10^, and lipoprotein P68^11^, are known to function as adhesins. However, despite these preliminary findings, the adherence of *M. bovis* to its host cells remains to be clarified.

Many highly conserved bacterial proteins involved in metabolic regulation or the cell stress response, such as α-enolase, have also been found to act as adhesins^12, 13^. Among these, NADH oxidase (NOX) of *Streptococcus pneumoniae* (*S. pneumoniae*) has recently been shown to display adhesion-related activity^14^. Because a homologue of the *nox* gene exists in the *M. bovis* genome, we hypothesized that this homologue might function as both an active enzyme and an adhesin in *M. bovis*. Our results confirm this hypothesis.

## Results

### Bioinformatic analysis, cloning, and expression of *M. bovis* NOX

**Figure 1:**
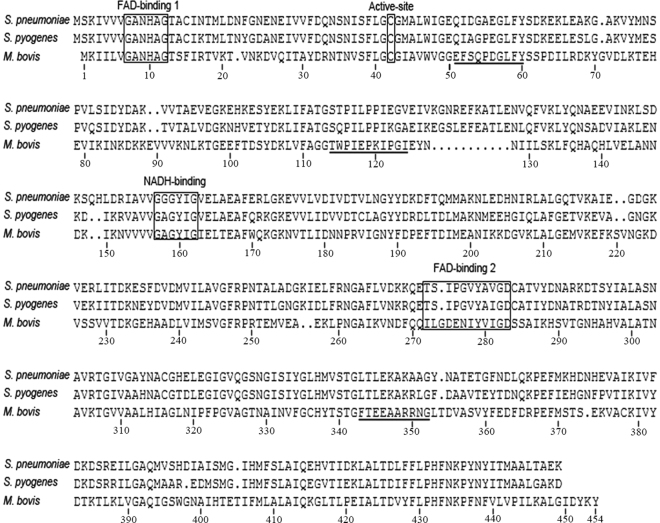
Comparative distribution of the motifs for enzymatic activity and B epitopes in the sequences of NADH oxidase (NOX) of *M. bovis* and *S. pyogenes*. The domains or sites for enzyme activity are indicated in box in *M. bovis* and *S. pyogenes* NOX. The B-cell epitopes predicted by BepiPred are underlined. The numbers are labeled according to the amino acid sequence of *M. bovis* NOX.

The full-length coding sequence (CDS) of *M. bovis nox* is 1365 bp, and the predicted protein contains 454 amino acids. A DNA alignment revealed similarities of 39% and 42% between the *M. bovis nox* gene and that of *S. pneumoniae* and *S. pyogenes*, respectively. When the putative *M. bovis* NOX protein was compared with the crystal structure of *S. pyogenes* NOX using DNAMAN, *M. bovis* NOX was found to have one catalytic residue (Cys 42), two FAD-binding domains (Gly 7–Gly 12; Ile 272–Asp 283), and a NADH-binding domain (Gly 157 to Gly 162) (Fig. 1). Interestingly, the amino acids in FAD-binding domain 1, the NADH-binding domain, and the active site are identical between the NOX proteins of *S. pyogenes* and *M. bovis*, indicating that they are highly conserved. However, the amino acids of FAD-binding domain 2 are quite different in these two species, although the terminal amino acids in this domain are the same, with the sequence “IXXIGD”, where X represents any amino acid (Fig. 1). The B-cell epitopes of NOX (Thr 114–Ile 124; Phe 343–Gly 352), predicted with a bioinformatic analysis, do not overlap any amino acid in the NADH-binding or the two FAD-binding sites or the conserved cysteine (Cys 42) (Fig. 1). Therefore, we hypothesized that the enzymatic activity of rNOX might not affect the interaction between rNOX and antibodies directed against it.

**Figure 2:**
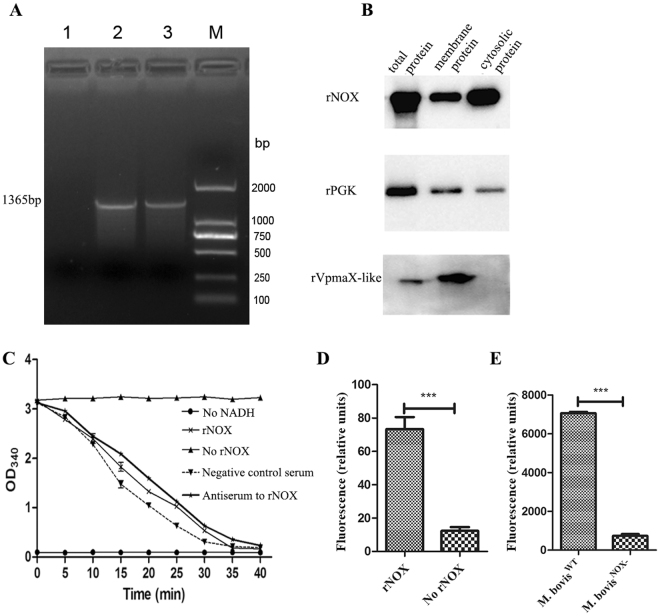
The cloning, expression, and enzymatic activity of NADH oxidase (NOX). (**A**) PCR amplification of *M. bovis nox* gene. Lane 1: negative control; Lane 2 and Lane 3: The mutated *nox* gene of *M. bovis*. M: DNA marker. (**B**) The localization of NOX and PGK in *M. bovis*. The total proteins, membrane proteins and cytosolic proteins were incubated with mAbs to rNOX (1:1000), mAbs to rVpmaX-like protein (1:1000), and antiserum against PGK (1:500). The membrane protein rVmapX-like served as positive control. The cropped blots are displayed here, the full-length blots are presented in supplementary information (Fig. S4). (**C**) Enzymatic activity of purified rNOX at 5 μg/ml. The OD_340 nm_ values of rNOX group decreased with the time increasing, whereas no reduction in absorbance was observed in negative control (No rNOX) and blank control (No NADH). 5 μl mouse antiserum to rNOX (1:200) incubated with rNOX before testing enzyme activity, while the equal amount of mixed negative serum served as negative control serum. Compared the antiserum to rNOX with negative control serum and rNOX, we found anti-rNOX polyclonal antibody cannot affect rNOX enzyme activity. (**D**) Enzymatic activity of rNOX. After rNOX converted NADH to NAD^+^, a kit was used to confirm whether rNOX produced H_2_O_2_. Compared to No rNOX, it was determined that rNOX could produce H_2_O_2_. (**E**) H_2_O_2_ production by *M. bovis* ^NOX−^ and *M. bovis* ^WT^ grown in glycerol as the carbon source. Statistical significance was determined by student t test (p < 0.001(***)).

The CDS of *M. bovis nox* (MBOV_RS01500) was cloned. The gene was modified (UGA → UGG) at eight sites and its correct insertion into pNOX was confirmed with PCR and DNA sequencing (Fig. 2A). The CDS was successfully expressed in *Escherichia coli* (*E. coli*) as a His-tagged soluble protein after induction with isopropyl β-d-thiogalactoside (IPTG). The purified recombinant protein rNOX was used to produce specific polyclonal and monoclonal antibodies (mAbs). The titers of mouse mAbs directed against rNOX or rVpmaX-like protein, which were determined with an indirect enzyme-linked immunosorbent assay (iELISA), were 10^6^. The titer of a mouse antiserum against recombinant phosphoglycerate kinase (rPGK) was 10^6^ either.

The reactivity of the anti-rNOX mAb was analyzed with a western blotting assay using different cellular fractions of *M. bovis*, including the total, membrane and cytosolic proteins. As shown in Fig. 2B, the anti-rNOX antibody reacted strongly with a 49-kDa protein in all the protein fractions, including *M. bovis* total proteins, cytosolic and membrane proteins, but reacted more strongly with the cytosolic proteins than with the membrane proteins. In contrast, the VpmaX-like membrane protein was only detected in the total protein and membrane protein of *M. bovis*. This result is consistent with that of a previous study^10^. The predictions made with the Signal IP and TMHMM servers illustrated that NOX has no transmembrane domain or signal peptide (Suppl. Fig. S1). Accordingly, when the mAbs directed against rNOX were incubated with the proteins extracted from the culture supernatant, no NOX reactive band was detected.

Similarly, the CDS of the *pkg* gene was modified (UGA → UGG) at three sites and confirmed with DNA sequencing. rPGK was successfully expressed and antiserum directed against rPGK recognized the PGK present in both the cytosolic and membrane fractions.

### *Mycoplasma bovis* NADH oxidase is an active enzyme

The enzymatic activity of rNOX was confirmed by detecting both the oxidation of NADH to NAD^+^ and reduction of O_2_ to H_2_O_2_. The rNOX (5 µg/ml) displayed NADH oxidative activity by converting NADH to NAD^+^ (Fig. 2C). In the absence of rNOX or NADH, no catalytic activity was observed. The enzymatic activity of rNOX was not influenced by anti-rNOX serum, suggesting that its sites for adhesion and catalysis are independent of one another (Fig. 2C). H_2_O_2_ was also produced in significantly higher quantities in the catalysis reaction system containing rNOX than in the blank control (No rNOX) (*p* < 0.001) (Fig. 2D). In addition, the H_2_O_2_ production by the mutant of NOX gene (*M. bovis* ^NOX−^) whose construction was described below was determined to be lower significantly than the wild type *M. bovis* HB0801 (Fig. 2E).

### rNOX binds EBL cells

**Figure 3:**
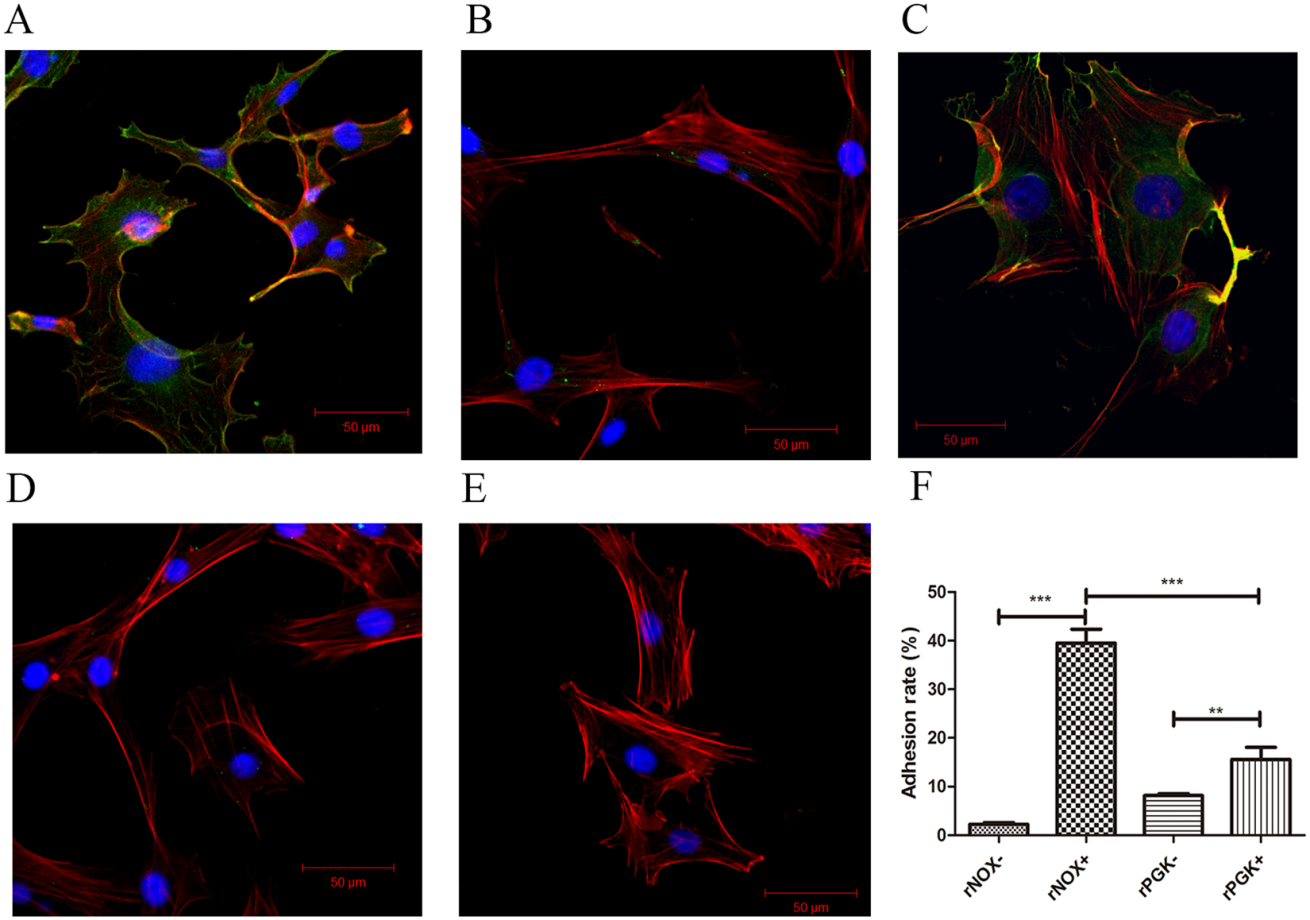
Adhesion and its inhibition of rNOX to EBL cells. Adhesion of rNOX to EBL cells. 10^6^ EBL cells interacted with 10 μg rNOX (**A**), 10 μg rPGK (**D**), or PBS (**E**). For adhesion inhibition of rNOX. 10 μg rNOX was pre-treated with 10 μl mouse antiserum to rNOX (**B**) or 10 μl mixed negative serum (**C**) in 1 ml MEM and then interacted with EBL cells. EBL cell actin filaments were labeled red with Rhodamine phalloidin and cell nuclei labeled blue with 4, 6-diamidino-2-phenylindole (DAPI). The rNOX was labeled green by mouse anti-rNOX mAb and goat anti-mouse IgG-FITC. (**F**) The adhesion and its inhibition of rNOX to EBL cells detected by flow cytometry. GraphPad Prism 5 software was used to evaluate the adhesion of rNOX to EBL. Compared with the rPGK by student t test, rNOX had a strong ability of adhesion to EBL cells (p < 0.001(***)).

Confocal laser scanning microscopy was used to visualize the adhesion of rNOX (green) to EBL cells whose F-actins were labeled with red. rNOX adhered strongly to the EBL cells, appearing as a merged yellow signal where rNOX co-localized with the cellular actins (Fig. 3A). The binding of rNOX to EBL cells was effectively inhibited by antiserum directed against rNOX (Fig. 3B), whereas negative serum from mock-immunized mice did not affect rNOX binding (Fig. 3C). In contrast, the unrelated *M. bovis* protein rPGK did not bind to the EBL cells (Fig. 3D). In the absence of rNOX in the blank control, the EBL cells showed no green fluorescence (Fig. 3E), indicating that no rNOX protein had bound. The differential binding of rNOX and rPGK to EBL cells was probed by mAb to rNOX and anti-serum to rPGK and quantitatively assayed for 10000 cells with flow cytometry and the results were consistent with the morphological observations. The adhesion rates of the two proteins, rNOX and rPGK, differed significantly (*p* < 0.001) (Fig. 3F and Suppl. Fig. S2).

**Figure 4:**
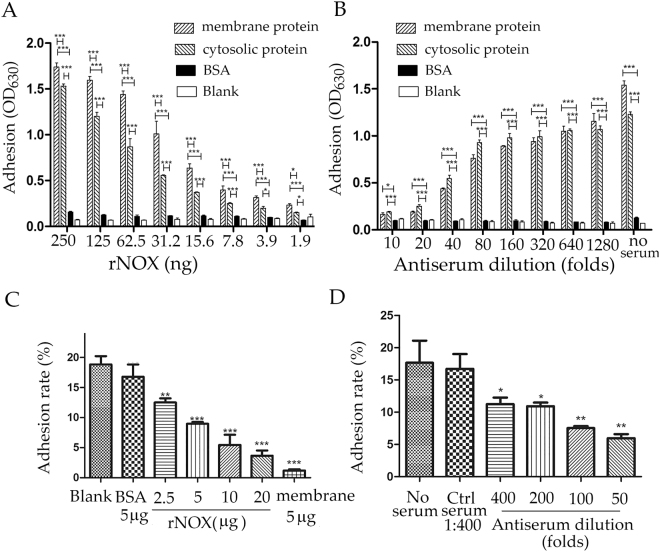
rNOX adhesion and adhesive inhibition assays with ELISA and flow cytometry. (**A**) Adhesion of rNOX to EBL cells detected by ELISA. 0.5 μg rNOX in 100 μl PBST containing 5% BSA was serially diluted to 128-folds and 100 μl of each dilution was added into the coated wells and incubated in the plates. (**B**) Adhesive inhibition of rNOX to EBL cells detected by ELISA. The adhesion of 0.25 μg rNOX was inhibited by serial dilutions (from 1:10 to 1:1280) of antiserum to rNOX, and the equal amount of rNOX treatment without antiserum served as control. The wells coated with BSA (0.5 μg/well) as the negative control or non-coated well as blank control. (**C**) The adhesion of *M. bovis* to EBL cells inhibited by rNOX. The 10^6^ EBL cells were incubated with different concentration of rNOX before infection. BSA (5 μg) in 1 ml of MEM, 5 μg of *M. bovis* membrane proteins in 1 ml of MEM, and 1 ml of MEM alone were used as the negative, positive, and blank controls, respectively. (**D**) The adhesion of *M. bovis* to EBL cells inhibited by anti-rNOX serum. *M. bovis* were incubated with anti-rNOX serum diluted from 1:50 to 1:400 before infection. The mixed negative serum (three unimmunized mice serum) was severed as negative control and 1 ml of MEM without serum (No serum) was used as the blank control. *p < 0.05, **p < 0.01, ***p < 0.001 represent statistically significant difference, and very significant difference, while “ns” represents no difference.

The interaction between rNOX and EBL cells was further characterized with an ELISA. The membrane and cytosolic proteins of the EBL cells were coated separately onto ELISA plates. rNOX bound dose-dependently to both the membrane and cytosolic proteins from epithelial cells (*p* < 0.05), but it bound significantly more strongly to the membrane proteins than to the cytosolic proteins (*p* < 0.05) (Fig. 4A). The binding of rNOX to both the EBL membrane and cytosolic proteins was significantly and concentration-dependently inhibited by antiserum directed against rNOX (*p* < 0.05). In contrast, neither bovine-serum albumin (BSA)-coated nor blank wells bound rNOX (Fig. 4B).

### Both rNOX and anti-rNOX serum inhibited *M. bovis* adhesion to EBL cells

The adhesion of *M. bovis* labeled with carboxyfluorescein diacetate succinimidyl ester (CFDA-SE) to EBL cells was analyzed with flow cytometry (Fig. 4C,D). To check the binding specificity of rNOX, rNOX was two-fold serially diluted using 1 ml MEM, corresponding to 2.5–20.0 μg/ml of protein. The EBL cells were pretreated with the different dilutions of rNOX and the binding between the EBL cells and *M. bovis* was determined*.* rNOX significantly and dose-dependently inhibited the binding of *M. bovis* to the cells (Fig. 4C and Suppl. Fig. S3). However, there was no significant difference in inhibition between the BSA control and the blank control groups (Fig. 4C and Suppl. Fig. S3).

The inhibitory effect of anti-rNOX serum was tested (Fig. 4D and Suppl. Fig. S3). Relative to the inhibitory effect of the negative serum on adhesion, all the anti-rNOX serum dilutions (from 1:50 to 1:400) dose-dependently inhibited the binding reaction to some extent. In contrast, there was no significant difference between the inhibition exerted by the negative serum or the blank control (Fig. 4D and Suppl. Fig. S3).

### *Mycoplasma bovis*^NOX−^ strain is deficient in NOX expression and binding ability

**Figure 5:**
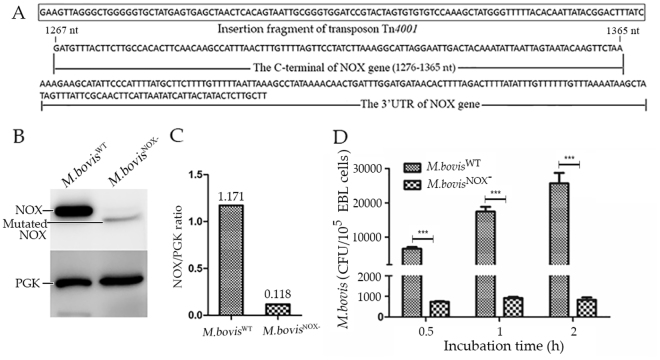
Adhesion of *M. bovis* ^NOX−^ to EBL cells. (**A**) The result of sequencing Tn4001. The terminal sequence of Tn4001 was used for the amplification of sequence next to it. Then the sequence was used to map transposon insertion site and confirmed the NOX gene was inserted at 1276 nt (C-terminal of NOX gene). (**B**) The expression of NOX in *M. bovis* ^NOX−^. The total proteins of *M. bovis* ^NOX−^ and *M. bovis* ^WT^ were incubated with antiserum against rPGK (1:500) or mAb (1:1000) to rNOX to detect the expression of NOX gene. The cropped blots are displayed here, the full-length blots are presented in supplementary information (Fig. S5). (**C**) Image J software was used to calculate the NOX/PGK ratio and then the expression of disrupted *nox* gene in *M. bovis* ^NOX−^ was compared with that intact *nox* in *M. bovis* ^WT^. (**D**) The adhesion rates of *M. bovis* ^NOX−^ at three time points with MOI of 1:1000. ***p < 0.001 represents very significant difference.

DNA sequencing showed that transposon Tn*4001* was inserted at the site of nucleotide 1276, causing transcription to stop 90 bp from the end of the gene (Fig. 5A). This insertion led to the expression of a truncated 45-kDa NOX protein at a significantly reduced level compared with expression of the intact 49-kDa NOX (Fig. 5B). A quantitative analysis with the ImageJ software, based on the expression of PGK, indicated that the expression of the truncated protein was reduced by 10-folds (Fig. 5C).

The adhesion ability of the *M. bovis* ^NOX−^ strain to EBL cells was tested with a plating assay and compared with the ability of the *M. bovis* ^WT^ strain after incubation for 30 min, 1 h, or 2 h in 24-well plates. The amount of *M. bovis* ^NOX−^ (CFU of recovered *M. bovis* ^NOX−^ per 10^5^ EBL cells) was less than 1000 at these three time points, and did not change noticeably as the incubation time increased. In contrast, the amount of recovered *M. bovis* ^WT^ increased significantly as the incubation time increased and the ratios of the average amount of *M. bovis* ^WT^ to the average amount of its mutant *M. bovis* ^NOX−^ at the three time points were 9, 19, and 30, respectively (Fig. 5D). The amounts of the two strains of *M. bovis* recovered differed significantly at all three time points (*p* < 0.001).

The ability of *M. bovis* ^NOX−^ to produce H_2_O_2_ was tested. As mentioned above, *M. bovis* ^NOX−^ produced significantly less H_2_O_2_ than *M. bovis* ^WT^ (*p* < 0.001) (Fig. 2E).

### Identification of rNOX binding ligands

**Figure 6:**
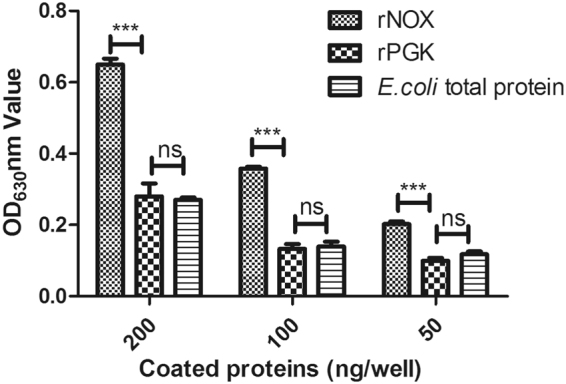
The fibronectin-binding ability of rNOX. The rNOX, rPGK, and *E. coli* total protein were applied to examine the fibronectin activity with indirect ELISA. Different amounts of proteins were coated in the 96 wells of ELISA plate. After washing and blocked with 5% BSA, 200 ng fibronectin in 100 μl PBST was added into each well and incubated for 1 h. And then the anti-fibronectin mAb and goat anti-mouse antibodies were sequentially overlaid to detect the binding activity. ***Represents very significant difference (*p* < 0.001), while “ns” represents no difference.

The T7 phages displaying human lung cDNA library were screened with rNOX coated onto wells in ELISA plate. After five rounds of biopanning, phages that bind to rNOX were significantly enriched shown by increasingly low ratio of titer of input phages to titer of retained phages (Fig. S6). Finally DNA sequences of 96 enriched phages after fifth panning were determined by PCR amplification and sequencing of the PCR product (Fig. S7). As a result, five sequences were identified (Table S1). The sequences were further aligned against the human genome (GenBank accession no: GCA_000001405.24) using online BlastX. The amyloid precursor-like protein-2 (APLP-2) and fibronectin (FN) were identified. Then, we compared the peptide sequences of APLP-2 and FN between human and bovines respectively and discovered they have 98% and 90% similarity at amino acid level. Since commercial FN protein and its mAb are available, the binding between FN and rNOX were preferentially examined with ELISA. The results indicated rNOX has a stronger binding ability to FN compared with the binding ability of unrelated proteins rPGK and *E. coli* total proteins (Fig. 6).

## Discussion

The adhesion of microorganisms, including mycoplasma bacteria, to their host cells is a crucial step in their colonization and subsequent infection of the host. *Mycoplasma bovis* is a long-known pathogen, but the molecular basis of its adhesion is still poorly understood^1^. Because *M. bovis* has no cell wall, the membrane and membrane-associated proteins are believed to play significant roles in its adhesion. In this study, using a mAb probe, we showed NOX to be a membrane-associated protein, with no extracellular presence in the culture supernatant. A bioinformatic analysis demonstrated that NOX has no signal peptide or transmembrane domain.

Using a bioinformatic analysis, we predicted that *M. bovis* NOX is an active NADH oxidative enzyme, like the corresponding proteins of *S. pyogenes* and *S. pneumoniae,* because it has similar enzyme domains^14^. A series of experiments confirmed the enzymatic activity of rNOX by its oxidation of NADH to NAD^+^ and reduction of O_2_ to H_2_O_2_. The expression of NOX in the mutant *M. bovis* ^NOX−^ was reduced 10-folds, and its enzymatic activities were reduced accordingly (*p* < 0.001). Therefore, *M. bovis* NOX is an active NADH oxidase.

As previously reported, the NOXs are a class of enzymes that are thought to detoxify molecular oxygen (O_2_)^15^. They catalyze the two-electron reduction of O_2_ to H_2_O_2_ or the four-electron reduction of O_2_ to H_2_O^16^. Therefore, the NOXs may contribute to a more general mechanism of adaptation to O_2_, allowing pathogen growth under oxidative stress^17^. Because NADH oxidase binds nucleic acids and causes their degradation by oxidizing NADH to produce H_2_O_2_, it might capture the host DNA and degrade it to provide a source of nucleotides, as previous study described in the Archaea^18^. Therefore, *M. bovi*s NOX may contribute to the adaption of the bacterium to the O_2_-enriched environment of the bovine respiratory tract and its access to nutrients for growth, such as nucleic acids.

Although there are strong similarities between the NOX proteins of *M. bovis*, *S. pyogenes*, and *S. pneumoniae*, unlike *M. bovis* NOX, the NOXs of *S. pyogenes* and *S. pneumoniae* are reported to reduce O_2_ to produce H_2_O rather than H_2_O_2_^19, 20^. This study is the first to demonstrate that *M. bovis* NOX plays an important role in the generation of H_2_O_2_. The capacity to produce H_2_O_2_ is recognized as a critical virulence factor in *M. mycoides* subspecies^21^, so NOX may also be a virulence factor in *M. bovis*, mediating H_2_O_2_ production.

The *E. coli* expression product rNOX was used to study the activity of NOX natural protein based on evidences from the previous studies. The activities of several homologous proteins of NADH oxidase from *Giardia lamblia* ^22^, *Lactobacillus brevis* ^23^, *Lactobacillus pentosus* ^24^, and *Streptococcus pneumonia* ^14^, are successfully studied in the forms of recombinant proteins expressed by *E. coli*. In this study, our results by using rNOX expressed by *E. coli* firmly indicate that rNOX of *M. bovis* can oxidize NADH to NAD^+^ and reduce O_2_ to H_2_O_2_ shown in Fig. 2C,D.

Meanwhile, we used several methods, including confocal microscopy, flow cytometry, and ELISA, to demonstrate that both rNOX and native NOX of *M. bovis* mediate bacterial adhesion to EBL cells. To exclude any nonspecific reaction of the recombinant protein in the experiment, we expressed *M. bovis* PGK as a nonadhesive membrane-associated protein. Its nonadhesive characteristics were discovered coincidently when we previously screened adhesion proteins for *M. bovis*. Besides, very fortunately, during the late stage of this study, one member of our team identified an *M. bovis* NOX mutant from a transposon library constructed by this laboratoy. This mutant made it possible to study the adhesion function of NOX within the context of intact *M. bovis*. A comparative study of wild type *M. bovis* and this NOX mutant drew the same conclusions as the rNOX-based study: that NOX mediates the binding of *M. bovis* to EBL cells. The expression of the truncated NOX protein in *M. bovis* ^NOX−^ was 10-folds lower than the expression of NOX in WT *M. bovis*, and the adhesion of the mutant was significantly reduced. Because the mutant *M. bovis* ^NOX−^ expressed a truncated NOX, 4 kDa smaller than the intact WT NOX, it will be necessary to determine whether it is this truncation or its reduced expression that contributes to the reduced adhesion of the mutant.

In addition, phage display techniques further identified the APLP2 and FN may be potential rNOX-binding ligands. Between them, FN has been more widely studied and determined a popular extracellular matrix (ECM) protein which bridges between the pathogens and host cellular receptors^25^. Several fibronectin-binding bacterial proteins were discovered to mediate adhesion and subsequent invasion of bacteria to host cells through binding to FN^26^, for example, SfbI of *S. pyogenes* ^27^ and Ssa of *S. suis* ^28^. Since the binding of rNOX to FN was confirmed *in vitro* (Fig. 6) and FN is conserved across human and bovine, it would be of significance to further verify this potential ligand in *M. bovis* and *in vivo*. In addition, APLP2 is an evolutionarily conserved ubiquitously expressed type I transmembrane protein. Interestingly it can bind to extracellular matrix proteins such as FN^29^. Since another possible ligand of rNOX determined by DNA sequencing is APLP2, it would be possible FN might function as the primary receptor of NOX and then APLP2 the second receptor to mediate *M. bovis* adhesion. The interaction between FN and APLP2 would help improve *M. bovis* adhesion. Of course further study should be performed to test this hypothesis.

In conclusion, this study demonstrates that *M. bovis* NOX is not only an active oxidase that can oxidize NADH to NAD^+^ and reduce O_2_ to produce H_2_O_2_, but also an adhesin that significantly mediates cell adhesion possibly through fibronectin. To the best of our knowledge, this is the first report of the double functions of *M. bovis* NOX, which may contribute to the virulence of *M. bovis*.

## Methods

### Ethics statement

All animal experiments were approved by the Experimental Animal Ethics Committee of Huazhong Agricultural University (permit number: SYXK(ER) 2015-0084) in strict accordance with the Hubei Regulations for the Administration of Affairs Concerning Experimental Animals issued in 2005.

### Bioinformatic analysis

The amino acid sequences of NOX from *M. bovis* HB0801 (protein accession no: WP_013954704.1), *S. pneumoniae* (protein accession no: WP_000036771.1), and *S. pyogenes* (protein accession no: WP_011184515.1) were retrieved from the National Center for Biotechnology Information database and the homologies among them were analyzed. We used the DNAMAN software to predict the active sites of *M. bovis* NOX by comparing it to the crystal structures of NOX (Protein Data Bank accession code: 2BC0) of *S. pyogenes* and *S. pneumoniae*. The linear B-cell epitopes of *M. bovis* NOX were predicted with BepiPred (http://www.cbs.dtu.dk/services/BepiPred). The cleavage sites of the signal peptides in NOX were predicted with the Signal IP Server (http://www.cbs.dtu.dk/services/SignalP/). The transmembrane helices in NOX were predicted with the TMHMM Server (http://www.cbs.dtu.dk/cgi-bin/webface2.fcgi?jobid=552344F10000183DA12F609F&wait=20).

### Bacterial strains, cell lines, and culture conditions

*Mycoplasma bovis* strain HB0801, isolated by this laboratory in Hubei Province, China, in 2008 and stored at the China Center for Type Culture Collection (CCTCC) (CCTCC no: M2010040), was used in this study. The strain was grown in complete pleuropneumonia-like organism (PPLO) medium (BD Company, Sparks, MD, USA), as described previously^30^.

The EBL cell line was kindly provided by Prof. Fei Xue of the National Engineering Research Center of Veterinary Biologics, Harbin Veterinary Research Institute, Chinese Academy of Agricultural Sciences, Harbin, China. The cells were grown in minimal essential medium (MEM) supplemented with 10% fetal bovine serum (Gibco, Grand Island, NY, USA), 100 U/ml penicillin, and 100 μg/ml streptomycin.

### Cloning and expression

The *M. bovis nox* gene (MBOV_RS01500) (GenBank accession no: AFM51667.1) was cloned from the *M. bovis* HB0801 genome (GenBank accession no: AFM52020.1) with overlapping PCR. To circumvent the specific translational barrier of the UGA codon of mycoplasmas in *E. coli*, the site-directed mutagenesis was used to alter the *M. bovis* UGA codon into UGG to ensure that *M. bovis* tryptophan was encoded in *E. coli*. To do so, we used overlapping PCR with the primer sets Pnox1/Pnox2, Pnox3/Pnox4, Pnox5/Pnox6, Pnox7/Pnox8, and Pnox9/Pnox10 (Table 1) and *Pfu* DNA polymerase (Thermo Fisher, Rockford, IL, USA). The full-length *nox* gene with the UGA → UGG modification at eight sites was cloned into the vector pET-30a (Novagen, Darmstadt, Germany) to generate the recombinant plasmid pNOX. The correct insertion of the *nox* gene into pNOX was confirmed with nucleotide sequencing (Sangon Company, Shanghai, China). rNOX was expressed in *E. coli* strain BL21(DE3) (TransGen Biotech, Beijing, China), and purified with nickel affinity chromatography (GE Healthcare, Piscataway, NJ, USA). Similarly, the *M. bovis* gene for phosphoglycerate kinase (*pgk*) (MBOV_RS03290) was modified (UGA → UGG) at three sites, by amplifying it from the *M. bovis* HB0801 genome with primer sets P669-1/P669-2, P669-3/P669-4, and P669-5/P669-6 (Table 2), and rPGK was expressed and purified with the method described above. Purified rNOX and rPGK were analyzed with electrophoresis (12% SDS-PAGE) and Coomassie Brilliant Blue staining.

**Table 1:** Oligonucleotide primers used for amplification and site-directed mutagenesis of NOX gene.

Primers	Sequence (5′ → 3′)	Sites in Nox gene (nt)	Notes for the underlined
Pnox 1	CCGGAATTCATGAAGATTATTTTAGTGGGAGCAAA	1–26	*Eco*R I site
Pnox 2	ATTCACCACCTACCCAAACTGC	133–154	UGA to UGG
Pnox 3	AGCAGTTTGGGTAGGTGGTGAAT	132–154	
Pnox 4	GTTCGATTGGCCATGTACCGC	335–355	
Pnox 5	GGCGGTACATGGCCAATCGAA	334–354	
Pnox 6	TTTTACCTTTTTGCCAGAATGCTTCAG	497–523	
Pnox 7	CTGAAGCATTCTGGCAAAAAGGTAAAA	497–523	
Pnox 8	GCGTTTCCCCATGAACCAAT	1180–1199	
Pnox 9	ATTGGTTCATGGGGAAACGCTATTCA	1180–1205	
Pnox 10	GACAAGCTTTTAATATTTGTAGTCAATTCCTAATGCC	1338–1365	*Hin*dIII site

**Table 2:** Oligonucleotide primers used for amplification and site-directed mutagenesis of PGK gene.

Primers	Sequence (5′ → 3′)	Sites in Nox gene (nt)	Notes for the underlined
P669-1	CAGGTACCATGAAAAAAAGTATTGATGAT	1–21	*Kpn* I site
P669-2	CAAATATATCACCCAATGATGCCCAATA	409–436	UGA to UGG
P669-3	ATTGGGTGATATATTTGTTAATGATGCTTTTGGAA	420–454	
P669-4	TTGGTCCATTCCACACAACTG	935–955	
P669-5	AAACAGTTGTGTGGAATGGACCAATG	932–957	
P669-6	ACGGATCCTTACTTATTTTCAATTGCACTA	1164–1185	*BamH* I site

### Production of mouse monoclonal and polyclonal antibodies

Ten female 4-week-old BALB/c mice were purchased from the China Hubei Provincial Center for Disease Control and Prevention, Wuhan, China, and raised in the animal facility of Huazhong Agricultural University to produce antibodies against three *M. bovis* proteins: rNOX, rPGK, and VmapX-like membrane protein (kindly provided by Miss Xi Chen). Briefly, the mice were immunized with a multipoint subcutaneous injection containing 100 μg of purified recombinant protein in 200 μl of phosphate-buffered saline (PBS) mixed with an equal volume of Freund’s complete adjuvant (Sigma–Aldrich, St. Louis, MO, USA). Two subsequent boosters, each with the same amount of protein in 200 μl of PBS together with an equal volume of Freund’s incomplete adjuvant (Sigma), were administered at intervals of 2 weeks. The antiserum titers were tested after each immunization. When the titers had increased significantly, the mice were euthanized, and the antiserum against each protein was collected. The spleen from one of the mice, immunized with either rNOX or rVmapX-like membrane protein, was then removed. The splenocytes were collected and used to produce mAbs against rNOX or rVmapX-like membrane protein, with a previously described procedure^31^. Briefly, the splenocytes from the immunized mice were harvested aseptically and fused with the myeloma cell line SP2/0 in a ratio of splenocytes to myeloma cells of 10:1, with 50% (w/v) polyethylene glycol 4000 (Sigma). The fused cells were cultured and selected in RPMI-1640 medium (Hyclone, Beijing, China) supplemented with 10% fetal bovine serum (Gibco), 100 μg/ml streptomycin, 100 U/ml ampicillin, and 2% hypoxanthine–aminopterin–thymidine (50×) (Sigma). The supernatants of the hybridoma cultures were screened with iELISAs, with each well of the ELISA plates precoated with 100 ng of rNOX or rVmapX-like. The positive hybridoma cells were cloned with limiting dilution, and single hybridoma clones stably secreting mAbs against either rNOX or rVmapX-like were selected. The hybridoma cells were injected into the abdominal cavities of BALB/c mice to generate tumors that produced ascites containing mAbs. The mAbs in the ascites were then purified with HiTrap™ Protein G HP (GE Healthcare, Piscataway, NJ, USA), and their immunological activities were determined with the conventional western blotting assay described below.

### Cellular localization of *M. bovis* NADH oxidase and PGK

The methods used to determine the localization of NOX and PGK in *M. bovis* have been described elsewhere^14^. In brief, the membrane and cytosolic proteins of *M. bovis* were extracted separately with a ProteoExtract® Transmembrane Protein Extraction Kit (cat. no. 71772-3) (Novagen, Darmstadt, Germany), according to the manufacturer’s instructions. The total cell proteins were prepared by sonicating the cells (200 W) on ice for 5 min. The protein concentrations were determined with a BCA kit (Cellchip Biotechnology Company, Beijing, China), and the protein samples were stored at −20 °C until use.

For the western blotting analysis, the membrane proteins (0.5 μg/lane), soluble cytosolic proteins (0.5 μg/lane), and total proteins in the cell lysates (0.5 μg/lane) were subjected to 12% SDS-PAGE and transferred to a nitrocellulose membrane (Millipore, Darmstadt, Germany). The rVmapX-like protein of *M. bovis* ^10^ was used as the positive control. The blotted membranes were cut into strips to separate the lanes and blocked with 5% skimmed milk powder in PBS containing 0.05% Tween 20 (PBST) at 4 °C overnight. The antibodies, including a mAb (diluted 1:1000) against rNOX, polyclonal antiserum against rPGK (1:500), and a mAb (1:1000) against rVmapX-like membrane protein, were added to the membrane strips, and the membranes were incubated at room temperature for 1 h. The membrane strips were washed three times with PBST and incubated for 1 h at room temperature with horseradish peroxidase (HRP)-conjugated goat anti-mouse IgG antibody (diluted 1:10,000) (Southern Biotech, Birmingham, MI, USA). After the membrane was washed three times, the protein bands on it were visualized with enhanced chemiluminescence (Thermo Fisher), according to the manufacturer’s protocol.

The presence of NOX in the culture supernatants was examined. Briefly, the proteins in the culture supernatant were extracted with a previously described method^32^, with slight modifications. *Mycoplasma bovis* was cultured in PPLO medium for 48 h and then centrifuged (15,400 × *g*, 20 min, 4 °C). The culture supernatant (100 ml) was then concentrated to 1 ml in an Amicon® Ultra-4 Centrifugal Filter Unit (15 ml, 10 kDa) (Millipore). To assess the presence of contaminating proteins, an equal volume of PPLO medium was concentrated as the negative control. After the protein concentrations in the culture supernatant and the control medium were determined with a BCA kit (Cellchip Biotechnology Company), 20 μg of protein from each sample was subjected to 12% SDS-PAGE and transferred to a nitrocellulose membrane (Millipore). The mAb against NOX was used to examine the localization of NOX with the method described previously. *Mycoplasma bovis* lysate (5 μg) was used as the positive control.

### Detection of rNOX enzymatic activity

The enzymatic activity of purified rNOX was determined by measuring the oxidation of NADH to NAD^+^, as described previously, with slight modification^23^. Briefly, for this enzyme assay we used 5 μg/ml rNOX, 20 mM potassium phosphate buffer (pH 7.0), 5 mM NADH (Sigma), 10 μM FAD (Sigma), 1 mM dithiothreitol, and no extra oxygen. rNOX was preincubated with FAD for 5 min and then incubated with NADH at 25 °C. The optical density (OD) at 340 nm (OD_340_) was measured at intervals of 5 min over a period of 50 min. To determine whether the adhesion site is independent of the enzymatic functional sites, we tested whether antiserum to rNOX, which blocked the adhesion of the protein to the host cells, reduced the enzymatic activity of rNOX. We preincubated rNOX with 5 μl of anti-rNOX serum (1:200) for 30 min at 37 °C and the enzymatic activity of rNOX was then tested under the conditions described above. Unimmunized mouse serum was used as the negative control serum. Reaction systems lacking the substrate NADH or rNOX were used as the blank (No NADH) or negative (No rNOX) control, respectively. The reduction in OD_340_ correlated positively with the enzymatic activity.

As well as converting NADH to NAD^+^, NOX can oxidize NADH to generate H_2_O_2_ or H_2_O. Therefore, the reaction system described previously, including rNOX and no rNOX, was used to determine the capacity of rNOX to produce H_2_O_2_. An H_2_O_2_ fluorometric assay, performed with the Hydrogen Peroxide Cell-Based Assay Kit (Cayman Chemical, Ann Arbor, MI, USA), was performed according to the manufacturer’s instructions, with slight modification. The catalysis reaction solution (8 μl) containing rNOX or the blank control was transferred to the wells of a 96-well plate, with each sample repeated in six wells. Assay buffer (72 μl) was added to each well to a total volume of 80 μl. Catalase solution (10 μl per well) was then added to half of the six wells in each group (control), and an equal amount of assay buffer was added to each of remaining three wells (sample). The enzyme reaction solution (10 μl per well) was then added to all the wells. The plate was then incubated for 20 min at room temperature with gentle shaking. The fluorescence intensity of each well (excitation at 530 nm; emission at 590 nm) was read with an ELISA plate reader (Winooski, USA). The adjusted fluorescence values for rNOX and no rNOX were calculated with the following formula:

Adjusted fluorescence = average fluorescence of wells with samples − average fluorescence of wells with controls.

The adjusted fluorescence values of rNOX and the blank control were compared. The experiments were performed independently three times.

### Observation of the adhesion of rNOX to EBL cells with confocal microscopy

EBL cells were propagated in MEM on microscope coverslips in six-well cell culture plates (Sigma) for 24 h. The cells were then fixed with 4% neutralized paraformaldehyde in PBS (pH 7.4) for 10 min and permeabilized with 0.5% Triton X-100 for 5 min at room temperature. The treated cells were blocked with 1% (w/v) BSA in PBS (pH 7.4) for 2 h at 37 °C.

For the adhesion assay, three groups were each assigned three wells. In the adhesion group, wells containing about 10^6^ cells on microscope coverglasses were incubated with 10 μg of rNOX in 1 ml of PBS for 1 h at 37 °C. In the blank group and negative control group, the cells were incubated with 1 ml of PBS and 10 μg of rPGK, respectively.

For the adhesion inhibition assay, two additional groups were included. In the blocking group, 10 μg of rNOX in 1 ml of PBS was preincubated with 10 μl of mouse antiserum to rNOX, and in its negative control, the protein was pretreated with an equal amount of normal serum pooled from three nonimmunized mice for 30 min at 37 °C. The treated rNOX was then added to the fixed cells.

The mouse anti-rNOX mAb (1:300) in PBS containing 1% BSA was added to the wells with rNOX in the adhesion and adhesion inhibition assays, and incubated overnight at 4 °C. The wells containing rPGK were probed with mouse antiserum against rPGK (1:100) and incubated overnight at 4 °C. After the cells were washed, the cells in all the wells were overlain with fluorescein isothiocyanate (FITC)-goat anti-mouse IgG antibody (H + L chain specific) diluted 1:1000 (Southern Biotech) and incubated at 37 °C for 1 h. Rhodamine phalloidin (100 nM) (Cytoskeleton, Denver, CO, USA) was used to label the polymerized form of actin at room temperature for 30 min. 4,6-Diamidino-2-phenylindole (DAPI) (Beyotime, Shanghai, China) was used to label the cell nuclei. The cells were washed with PBS between each step. Immunofluorescence was detected with an Olympus FV1000 laser scanning confocal microscope (Olympus FV1000 and IX81, Tokyo, Japan). The experiment was performed independently three times.

### Batch assay of rNOX adhesion to EBL cells using flow cytometry

EBL cells (10^6^) were incubated with 10 μg of rNOX or rPGK in 1 ml of PBS for 1 h at 37 °C. Cells incubated with PBS with no added protein were used as the blank control. After the cells were washed, mouse anti-rNOX mAb (1:300) in PBS or antiserum against rPGK (1:300) was added to the wells containing either rNOX or rPGK, respectively, and to the corresponding control wells, and incubated for 30 min at 37 °C. After the cells were washed three times, a goat anti-mouse IgG antibody (H + L chain specific)–FITC (1:1000) (Southern Biotech) was added to each well and incubated for 30 min at 37 °C. The ability of rNOX to bind to EBL cells was then evaluated with flow cytometry (BD FACSCalibur flow cytometer, BD, NJ, USA).

### Assay of rNOX adhesion to EBL cellular fractions with ELISA

The 96-well ELISA plates were coated at 4 °C overnight with protein (0.5 μg/well): either the cellular membrane or cytosolic fraction of EBL cells or BSA in sodium bicarbonate–sodium carbonate buffer (pH 9.6). The wells were then blocked with 5% BSA in PBST at 37 °C for 2 h and washed three times with PBST.

For the adhesion assay, 250 ng of rNOX in 100 μl of PBST was two-fold serially diluted to 128-folds and applied to the wells of ELISA plates. Each dilution was tested in triplicate in each group.

For the adhesion inhibition assay, antiserum against rNOX (2 mg/ml) was two-fold serially diluted from 1/10 to 1/1280, and 100 μl of each dilution was preincubated (in triplicate) with 250 ng of rNOX in 100 μl of PBST at 37 °C for 1 h. Each mixture (100 μl) was then added to the coated wells. An equal amount of rNOX in 100 μl of PBST was used as the control. The reactions proceeded at 37 °C for 1 h. After the wells were washed, the bound rNOX was detected by incubation with anti-rNOX mAb (1:3000 in PBST) at 37 °C for 1 h, and then with HRP-conjugated goat anti-mouse IgG antibody (1:20,000 in PBST) (Southern Biotech) at 37 °C for 30 min. The reaction was developed with the substrate tetramethylbenzidine/H_2_O_2_ (Wuhan Keqian Animal Biological Products Co., Ltd, Wuhan, China) and stopped with hydrogen fluoride. The OD_630_ was determined with an ELISA microplate reader (Bio-Tek) (Winooski, Chicago, USA).

### Assay of NOX role in *M. bovis* adhesion with flow cytometry

The adhesion of *M. bovis* to EBL cells was first tested. Briefly, *M. bovis* was cultured in a 5% CO_2_ atmosphere at 37 °C for 36 h and fluorescently labeled by incubation with 5 μM CFDA-SE at 37 °C for 30 min. The bacteria were washed to remove excess CFDA-SE, and *M. bovis* was counted with the plating assay. EBL cells (10^6^) were infected with fluorescently labeled *M. bovis* at 37 °C for 30 min at multiplicity of infection (MOI) of 1:1000. The cells were evaluated with flow cytometry (FACSCalibur flow cytometer) and the adhesion rate of *M. bovis* was assayed based on the fluorescence intensity of 10^4^ EBL cells.

The inhibition of adhesion by both rNOX and antiserum against rNOX was assayed. For the rNOX inhibition assay, rNOX was two-fold serially diluted and 10^6^ EBL cells were pretreated with each dilution of rNOX (2.5–20 μg) added to 1 ml of MEM before they were mixed with *M. bovis.* BSA (5 μg) in 1 ml of MEM, 5 μg of *M. bovis* membrane proteins in 1 ml of MEM, and 1 ml of MEM alone were used as the negative, positive, and blank controls, respectively. These experiments were performed independently three times.

To assay the inhibition by antiserum, *M. bovis* was preblocked with 5 μl (2 mg/ml) of antiserum to rNOX in 1 ml of MEM, diluted from 1:50 to 1:400, at 37 °C for 30 min, and then incubated with 10^6^ EBL cells at 37 °C for 30 min. The unbound *M. bovis* was removed by centrifugation at 1000 × *g* for 5 min. Serum (5 μl) from unimmunized mice in 1 ml of MEM was used as the negative control and 1 ml of MEM was used as the blank control.

### Assay of deficient NOX expression in *M. bovis*^NOX−^

The pMT85 plasmid containing the transposon Tn*4001*, kindly provided by Dr. Eric Baranowski (INRA, Toulouse, France), was randomly integrated into the *M. bovis* chromosome^33^. A random mutation library was constructed and individual mutants were determined with PCR using primers specific to the inserted Tn*4001* tag and DNA sequencing, in this laboratory. The mutant *M. bovis* ^NOX−^ was identified, in which the *nox* gene was knocked out by transposon insertion. The disruption of the *nox* gene was confirmed with PCR and DNA sequencing.

A western blotting assay was performed to confirm the absence of NOX expression in *M. bovis* ^NOX−^. The *M. bovis* wild type strain (*M. bovis* ^WT^) and its mutant strain *M. bovis* ^NOX−^ were cultured in PPLO medium containing gentamicin (400 μg/ml) for 48 h. The total cell proteins of both strains (*M. bovis* ^WT^ and *M. bovis* ^NOX−^) were prepared by sonication (200 W) on ice for 5 min. The proteins were then separated with SDS-PAGE (10%) and transferred onto a nitrocellulose membrane (Millipore). The membranes were treated with a mAbs (1:1000) directed against rNOX or antiserum directed against rPGK (1:500) (used as the negative control), and incubated at room temperature for 1 h. The membrane was then incubated with HRP-conjugated goat anti-mouse IgG antibody (1:10,000) for 1 h at room temperature. The bands on the membrane were visualized with enhanced chemiluminescence (Thermo Scientific), according to the manufacturer’s protocol. Between each two steps in the process, the membrane was washed three times with PBST. The ImageJ software was used to calculate the grayscale used to evaluate the differential NOX expression.

### Assay of adhesion and H_2_O_2_ production by *M. bovis*^NOX−^

The adhesion ability of *M. bovis* ^NOX−^ was determined with a plating assay. Briefly, the MOI was set at 1:1000 and the adhesion rates were measured at three time points (30 min, 1 h, and 2 h) after EBL cells were infected with either *M. bovis* ^WT^ or *M. bovis* ^NOX−^. The cells (10^5^ per well) were infected with 10^8^ CFU of both bacterial strains in 200 μl of complete MEM for the stated times. The cells were then washed five times with PBS, trypsinized, and collected by centrifugation at 500 × *g*. The cell pellet was 10-fold serially diluted with PPLO broth and 10 μl of each dilution was plated onto PPLO agar in Petri dishes to count the adherent mycoplasma. Each sample was tested in triplicate and the experiment was repeated independently three times.

The ability of *M. bovis* ^NOX−^ to produce H_2_O_2_ was determined as previously described^34^. *M. bovis* cultures were grown to mid-log phase, and the bacterial concentrations were estimated based on the OD_450_. *M. bovis* ^WT^ and *M. bovis* ^NOX−^ (1 × 10^8^ CFU) were collected by centrifugation at 10,000 × g for 10 min at 4 °C. The cells were then washed three times in 1 ml of HEPES buffer (67.6 mM HEPES, 140 mM NaCl, 7 mM MgCl_2_, pH 7.3), resuspended in 1 ml of HEPES, and incubated for 30 min at 37 °C. Glycerol was then added to each culture at a final concentration of 10 mM. After 20 min, an H_2_O_2_ fluorometric assay was performed with the Hydrogen Peroxide Cell-Based Assay Kit (Cayman Chemical), according to the manufacturer’s instructions, with slight modifications, as described above.

### Identification of rNOX-binding ligands

The commercial T7 Select^®^ human lung cDNA library (Novagen, Darmstadt, Germany) was applied to identify the rNOX binding sequences as described by the product’s manual. The rNOX (200 ng/well) was coated in 96 wells in an ELISA plate and interacted with phage. After 5 rounds of biopanning, the enriched phages were pated on TB agar plates. The 96 isolated plaques grown in liquid culture and amplified with the T7SelectUP and T7SelectDOWN primers. Then the PCR products were sequenced by the company (Sangon Company, Shanghai, China) and the corresponding genes were determined by using online BlastX.

Further, the rNOX, the negative control proteins rPGK, and total protein of *E. coli* containing pET-30a plasmid were two-fold serially diluted from 2 μg/ml to 0.5 μg/ml. For each dilution 100 μl were coated (in triplicate) in 96 well plate. The wells were then blocked with 5% BSA in PBST at 37 °C for 1 h and washed three times with PBST. 200 ng of fibronectin (Sigma) in 100 μl of PBST was applied to the wells of ELISA plates. The reactions proceeded at 37 °C for 1 h. After the wells were washed, the bound fibronectin was detected by incubation with anti-fibronectin mAb (Sigma) (1:1000 in PBST) at 37 °C for 1 h, and then with HRP-conjugated goat anti-mouse IgG antibody (1:5,000 in PBST) (Southern Biotech) at 37 °C for 45 min. The reaction was developed with the substrate tetramethylbenzidine/H_2_O_2_ (Wuhan Keqian Animal Biological Products Co., Ltd, Wuhan, China) and stopped with hydrogen fluoride. The OD_630_ was determined with an ELISA microplate reader (Bio-Tek) (Winooski, Chicago, USA).

### Statistical analysis

The data are expressed as means ± SD in the adhesion and adhesion inhibition assays. Student’s *t* test and ANOVA were performed with the software package in GraphPad Prism version 5 (La Jolla, CA, USA). Differences were considered statistically significant at *p* < 0.05 (*) or very significant at *p* < 0.01 (**) and *p* < 0.001 (***).

## Electronic supplementary material

Supplementary Information

## References

[1] Caswell JL, Archambault M (2008). *Mycoplasma bovis* pneumonia in cattle. Anim. Health Res. Rev..

[2] Caswell JL (2010). *Mycoplasma bovis* in respiratory disease of feedlot cattle. Vet. Clin. North Am. Food Anim. Pract..

[3] Maunsell FP, Donovan GA (2009). *Mycoplasma bovis* Infections in Young Calves. Vet. Clin. North Am. Food Anim. Pract..

[4] White BJ (2010). Mollicutes species and Mycoplasma bovis prevalence and association with health outcomes in beef feeder calves at arrival and initial treatment for bovine respiratory disease. Can. Vet. J..

[5] Wise KS (2011). Complete genome sequence of *Mycoplasma bovis* type strain PG45 (ATCC 25523). Infect. Immun..

[6] Li Y (2011). The complete genome sequence of *Mycoplasma bovis* strain Hubei-1. PLoS One.

[7] Qi JJ (2012). Comparative geno-plasticity analysis of *Mycoplasma bovis* HB0801 (Chinese isolate). PLoS One.

[8] Adamu JY (2013). Membrane proteins of *Mycoplasma bovis* and their role in pathogenesis. Res. Vet. Sci..

[9] Sachse K (1996). Mechanisms and factors involved in *Mycoplasma bovis* adhesion to host cells. Zentralbl Bakteriol..

[10] Zou X (2013). Molecular Cloning and Characterization of a Surface-Localized Adhesion Protein in *Mycoplasma bovis* Hubei-1 Strain. PLoS One.

[11] Lysnyansky I, Yogev D, Levisohn S (2008). Molecular characterization of the *Mycoplasma bovis* p68 gene, encoding a basic membrane protein with homology to P48 of Mycoplasma agalactiae. FEMS Microbiol. Lett..

[12] Brian H, Andrew M (2011). Bacterial Virulence in the Moonlight: Multitasking Bacterial Moonlighting Proteins Are Virulence Determinants in Infectious Disease. Infect. Immun..

[13] Song Z (2012). α-Enolase, an adhesion-related factor of Mycoplasma bovis. PLoS One.

[14] Muchnik L (2013). NADH oxidase functions as an adhesin in Streptococcus pneumoniae and elicits a protective immune response in mice. PLoS One.

[15] Higuchi M (1992). Reduced nicotinamide adenine dinicleotide oxidase involvement in defense against oxygen toxicity in Streptococcus mutans. Oral. Microbiol Immunol..

[16] Shi XC (2016). A water-forming NADH oxidase regulates metabolism in anaerobic fermentation. Biotechnol. Biofuels..

[17] Baolei J (2010). An Archaeal NADH Oxidase Causes Damage to Both Proteins and Nucleic Acids under Oxidative Stress. Mol. Cells..

[18] Minion FC (1993). Membrane-associated nuclease activities in mycoplasmas. J. Bacteriol..

[19] Mallett TC, Claiborne A (1994). Oxygen reactivity of an NADH oxidase C42S mutant: evidence for a C(4a)-peroxyflavin intermediate and a rate-limiting conformational change. Biochemistry..

[20] Auzat I (1999). The NADH oxidase of Streptococcus pneumoniae: its involvement incompetence and virulence. Mol. Microbiol..

[21] Pilo P (2005). A Metabolic Enzyme as a Primary Virulence Factor of Mycoplasma mycoides subsp. mycoides Small Colony. J. Bacteriol..

[22] Castillo-Villanueva A (2016). Cloning, Expression and Characterization of Recombinant, NADH Oxidase from Giardia lamblia. Protein J..

[23] Geueke B, Riebel B, Hummel W (2003). NADH oxidase from Lactobacillus brevis: a new catalyst for the regeneration of NAD. Enzyme Microb. Tech..

[24] Zhang J (2016). Cloning and characterization of two distinct water-forming NADH oxidases from Lactobacillus pentosus for the regeneration of NAD. Bioprocess Biosyst. Eng..

[25] Schwarz-Linek U, Hook M, Potts JR (2004). The molecular basis of fibronectin-mediated bacterial adherence to host cells. Mol. Microbiol..

[26] Norris NC (2011). Structural and functional analysis of the tandem beta-zipper interaction of a Streptococcal protein with human fibronectin. J. Biol. Chem..

[27] Towers RJ (2003). Evolution of sfbI Encoding Streptococcal Fibronectin-Binding Protein I: Horizontal Genetic Transfer and Gene Mosaic Structure. J. Clin. Microbiol..

[28] Li W, Wan Y, Tao Z, Chen H, Zhou R (2013). A novel fibronectin-binding protein of Streptococcus suis serotype 2 contributes to epithelial cell invasion and *in vivo* dissemination. Vet. Microbiol..

[29] Cousins SL, Dai W, Stephenson FA (2015). APLP1 and APLP2, members of the APP family of proteins, behave similarly to APP in that they associate with NMDA receptors and enhance NMDA receptor surface expression. J. Neurochem..

[30] Zhang H (2016). *Mycoplasma bovis* MBOV_RS02825 encodes a putative virulence-related nuclease. Int. J. Mol. Sci..

[31] Sanchez P (2014). Generating a Battery of Monoclonal Antibodies against Native Green Fluorescent Protein for Immunostaining, FACS, IP, and ChIP Using a Unique Adjuvant. Monoclon. Antib. Immunodiagn Immunother..

[32] Rebollo (2012). Extracellular Proteins of Mycoplasma synoviae. ISRN Vet. Sci..

[33] Skapski A (2011). Genome-scale analysis of Mycoplasma agalactiae loci involved in interaction with host cells. PloS one.

[34] Szczepanek SM (2014). Hydrogen Peroxide Production from Glycerol Metabolism Is Dispensable for Virulence of Mycoplasma gallisepticum in the Tracheas of Chickens. Infect. Immun..

