# Supplementary material for: Mycoplasma bovis NADH oxidase functions as both a NADH oxidizing and O2 reducing enzyme and an adhesin
Source: Sci Rep. 2017 Mar 3;7:44. doi: 10.1038/s41598-017-00121-y (PMC5427908; doi:10.1038/s41598-017-00121-y)
Supplement: Supplementary file 1 — Supplementary Information [file 41598_2017_121_MOESM1_ESM.pdf]

**Supplementary Information**

*Mycoplasma bovis* NADH oxidase functions as both a NADH oxidizing and $O_2$ reducing enzyme and an adhesion

Gang Zhao, Hui Zhang, Xi Chen, Xifang Zhu, Yusi Guo, Chenfei He, Farhan Anwar Khan, Yingyu Chen, Changmin Hu, Huanchun Chen, Aizhen Guo.

## SUPPLEMENTARY FIGURES

**Supplementary Figure S1. The localization of NOX in *M.bovis*.** (A) The detection of NOX in the culture supernatant. 20 μg proteins in the culture supernatant (lane 2), equal amount of proteins in the medium (lane 3) severed as negative control, and 5μg total proteins of *M.bovis* (lane 1) severed as positive control were incubated with mAb to NADH oxidase (1:1000). (B) The result of the prediction of signal peptide cleavage sites. (C) The result of the prediction of transmembrane helices by the TMHMM Server.

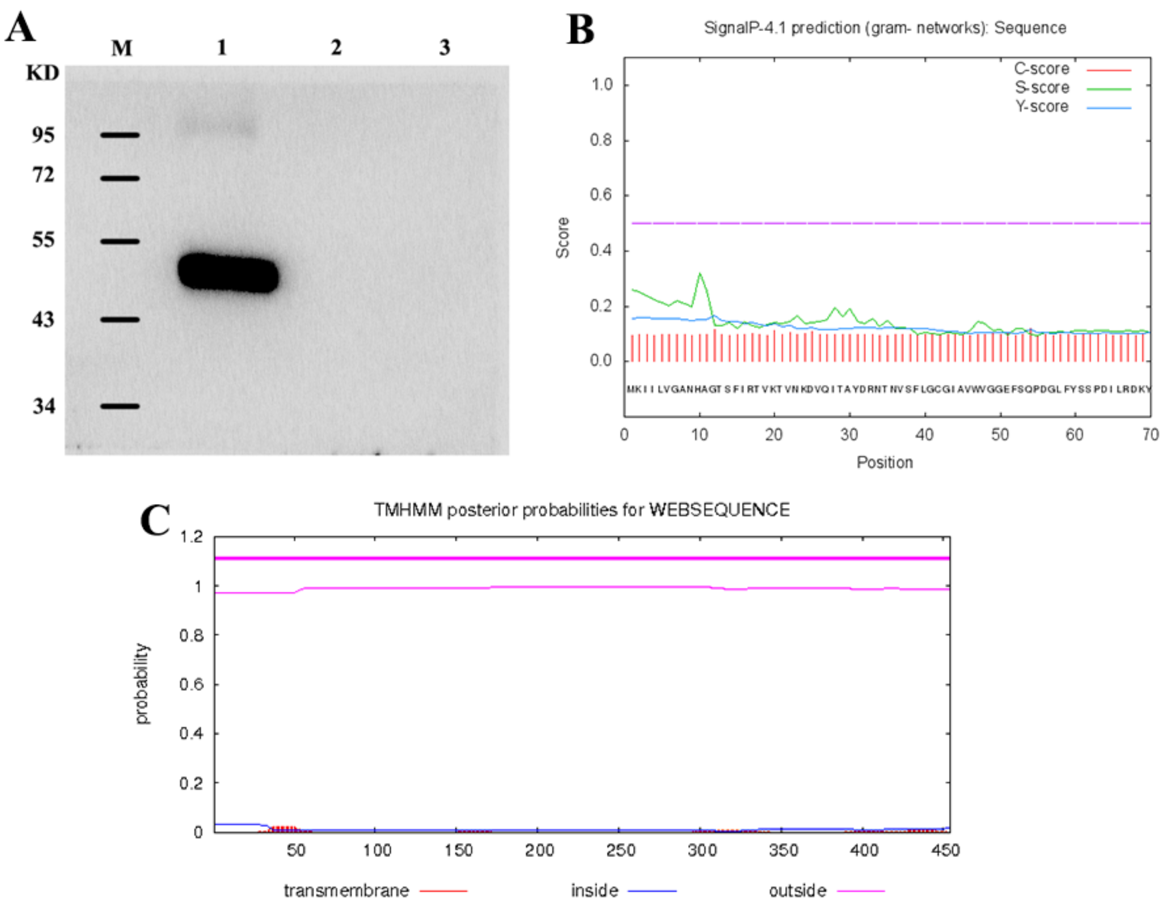

**Supplementary Figure S2. Adhesion assay of rNOX to EBL cells with flow cytometry.** The dot plots of $10^6$ EBL cell incubated with 10 μg rNOX or rPGK for 1 h at 37 ℃, and the cells without protein were served as control. Then, the cells without protein and cells incubated with rNOX or rPGK were overlain with antiserum against rPGK or mAb to rNOX respectively for 30 min at 37 ℃. The goat anti-mouse IgG-FITC was used to detect the adhesion of cells binding rNOX (rNOX+) or PBS (rNOX-) incubated with mAb, and the adhesion of cells binding rPGK (rPGK+) or PBS incubated with antiserum against rPGK. Each group had three repetitions.

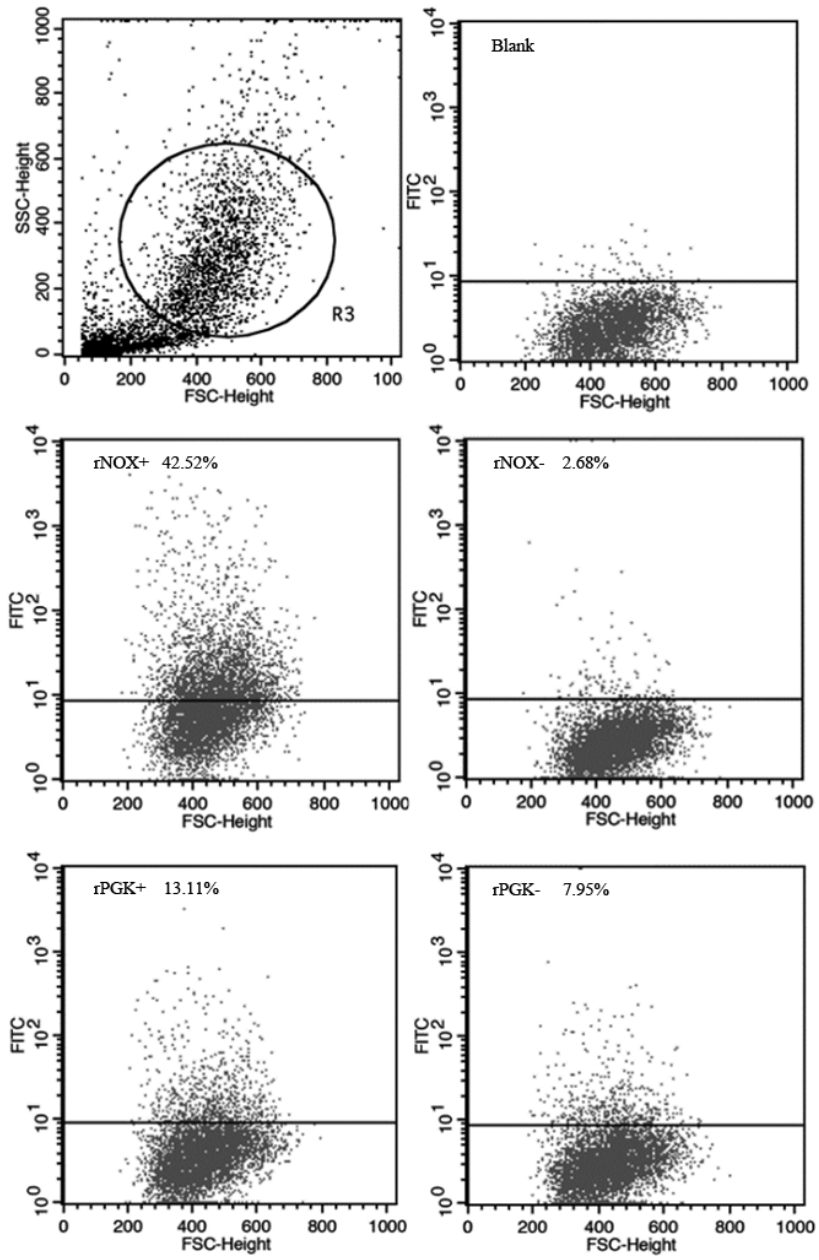

**Supplementary Figure S3. Adhesive inhibition of *M. bovis* to EBL cells detected by flow cytometry.** The $10^6$ EBL cells were infected with *M. bovis* (MOI=1000) for 30 min. (A) The adhesion inhibition with different amount of rNOX. The BSA and *M. bovis* membrane protein were used as negative control and positive control. (B) The adhesion inhibition with the anti-rNOX serum diluted from 1:50 to 1:400. The mixed negative serum (from sera of three non-immunized mice) was severed as negative control. The group without serum and that without additional proteins represented controls without treatments before incubation of *M. bovis* and the EBL cells.

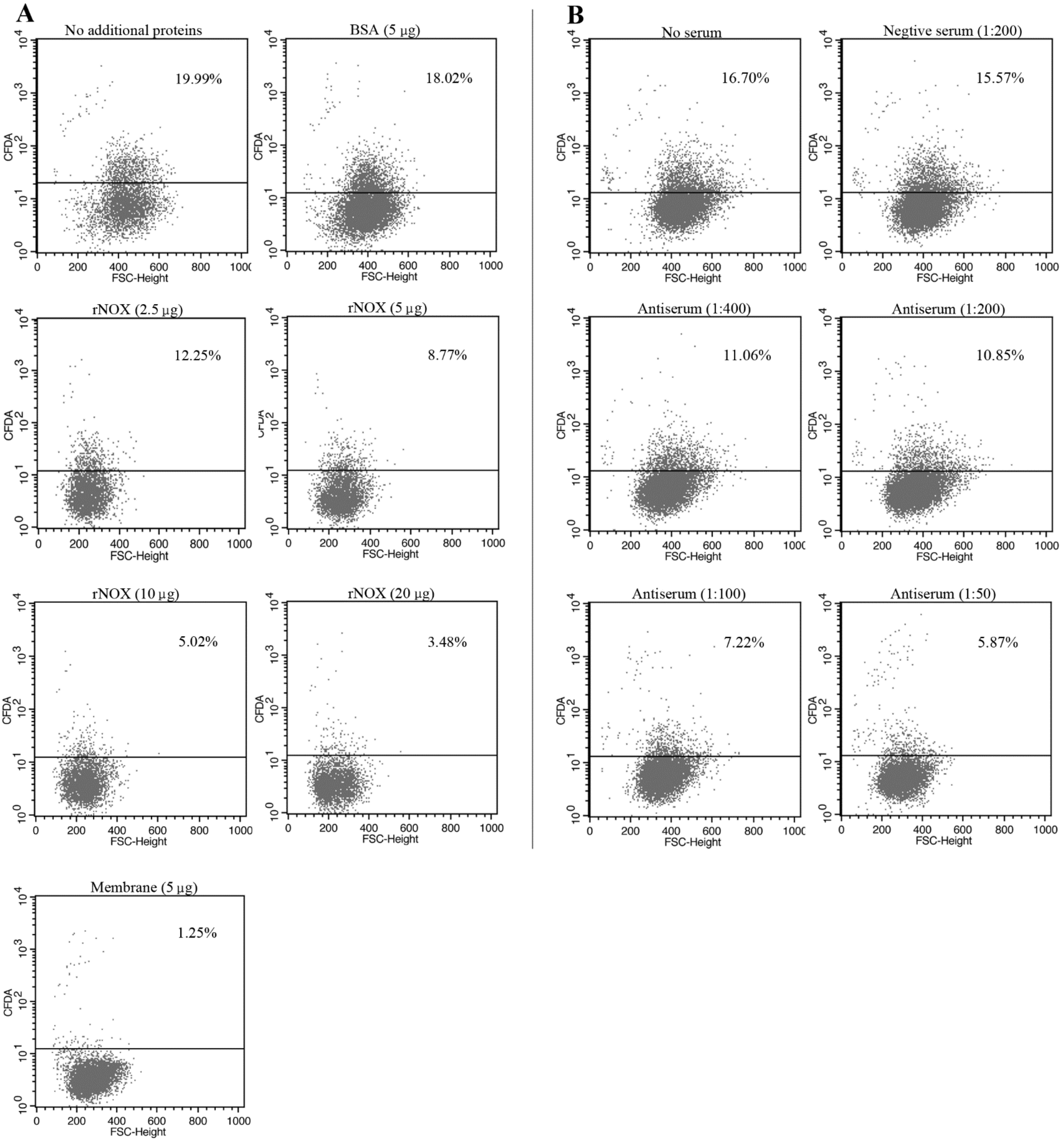
A
No additional proteins
19.99%
BSA (5 μg)
18.02%
rNOX (2.5 μg)
12.25%
rNOX (5 μg)
8.77%
rNOX (10 μg)
5.02%
rNOX (20 μg)
3.48%
Membrane (5 μg)
1.25%
B
No serum
16.70%
Negtive serum (1:200)
15.57%
Antiserum (1:400)
11.06%
Antiserum (1:200)
10.85%
Antiserum (1:100)
7.22%
Antiserum (1:50)
5.87%
CFDA
FSC-Height

**Supplementary Figure S4. The western blots illustrated the localization of NOX.** The total proteins (Lane 1), membrane proteins (Lane 2) and cytosolic proteins (Lane 3) were incubated with mAbs to rNOX (A), antiserum against PGK (B), and mAbs to rVpmaX-like protein (C).

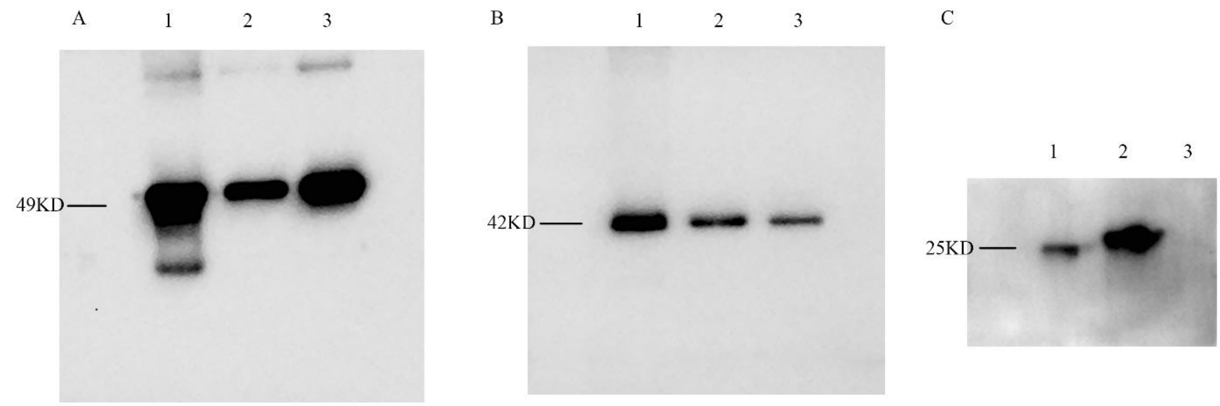

**Supplementary Figure S5. The expression of NOX in *M. bovis*$^{NOX-}$ detected by western blot assay.** Lane 1: the total proteins of *M. bovis*$^{WT}$; Lane 2: the total proteins of *M. bovis*$^{NOX-}$. (A) The western blot of mAb to rNOX detecting the expression of NOX gene. (B) The PGK regarded as control detected using antiserum against rPGK.

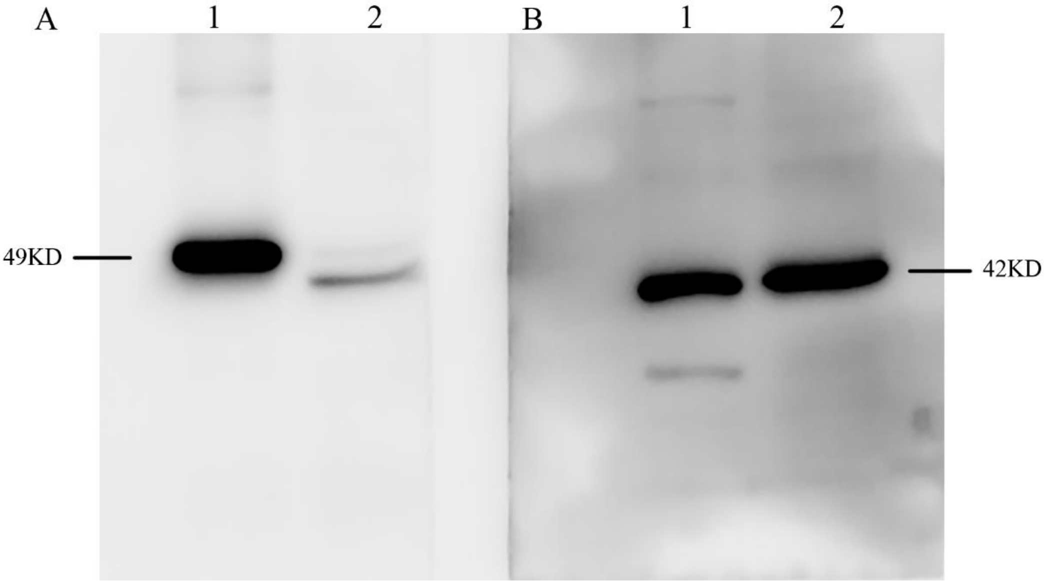


**Supplementary Figure S6. The result of screening the library by biopanning.** The ratio is equal to the titer of input phages/titer of retained phages. After 5 rounds of biopanning, the phages binding to rNOX were enriched.

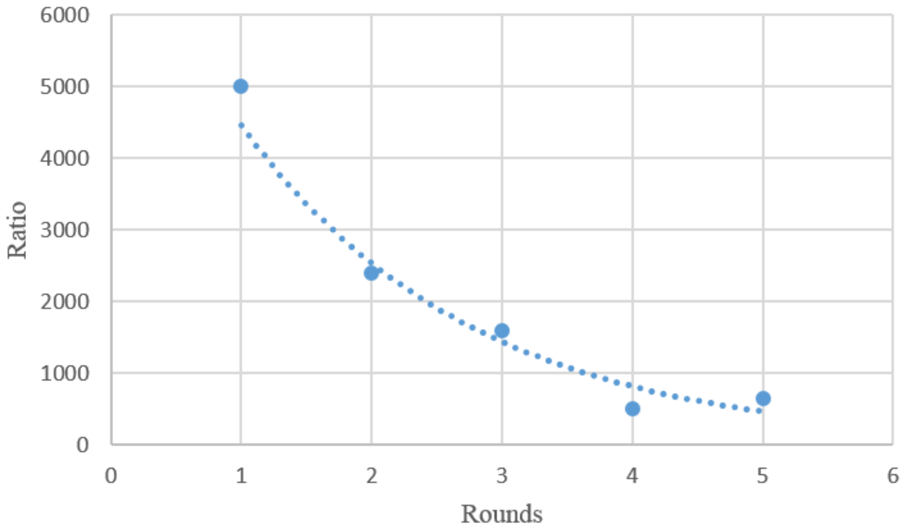

**Supplementary Figure S7. The results of PCR amplification of 96 plaques.** Every plaque amplified with the T7SlectUP and T7SelectDOWN primers illustrated the region surrounding the multiple cloning site.

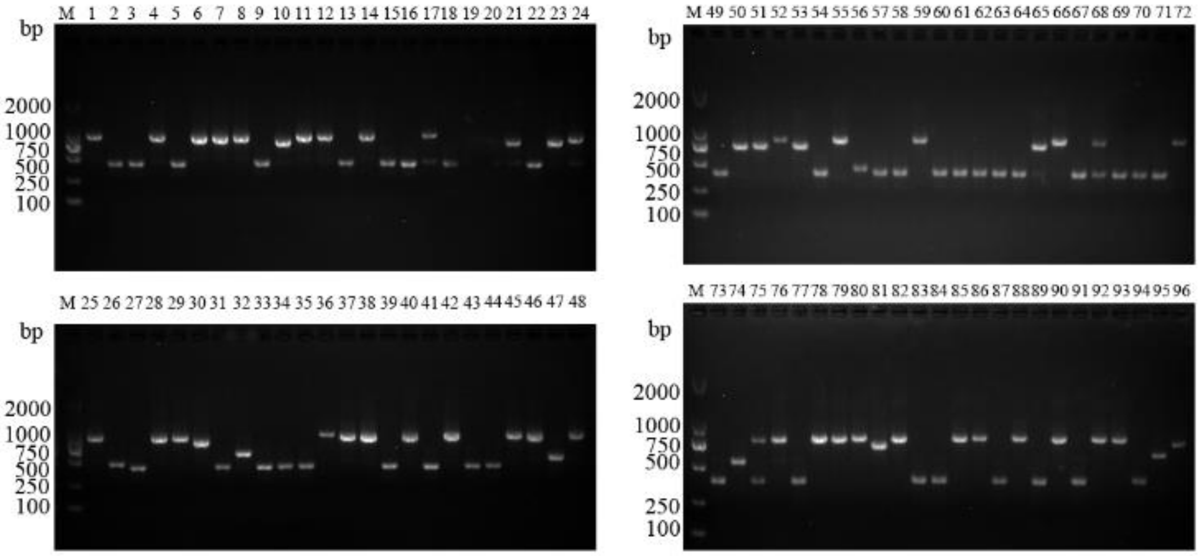

**Supplementary Table S1.** The five EST sequences by sequencing the PCR products.

| EST sequences | Predicted proteins (homology between human and bovine) |
|---|---|
| GGAGACTGATCGGTGCCGAAGAGAAAGTGATTAACAGTAAG<br>AATAAAGTGGATGAAAACATGGTCATTGACGAGACTCTGGAT<br>GTTAAGGAAATGATTTTCAATGCCGAGAGAGTTGGAGGCCTC<br>GAGGAAGAGCGGGAATCCGTGGGCCCACTGCGGGAGGACTT<br>CAGTCTGAGTAGCAGTGCTCTCATTGGCCTGCTGGTCATCGC<br>AGTGGCCATTGCCACGGTCATCGTCATCAGCCTGGTGATGCT<br>GAGGAAGAGGCAGTATGGCACCATCAGCCACGGGATCGTGG<br>AGGTTGATCCAATGCTCACCCCAGAAGAGCGTCACCTGAAC<br>AAGATGCAGAACCATGGCTATGAGAACCCCACCTACAAATAC<br>CTGGAGCAGATGCAGATTTAGGTGGCAGGGAGCGCGGCAGC<br>CCTGGCGGAGGGATGCAGGTGGGCCGGAAGATCCCACGATT<br>CCGATCGACTGCCAAGCAGCAGCCGCTGCCAGGGGCTGCGT<br>CTGACATCCTGACCTCCTGGACTGTAGGACTATATAAAGTACT<br>ACTGTAGAACTGCAATTTCCATTCTTTTAAATGGGTGAAAAAT<br>GGTAATATAACAATATATGATATATAAACCTTAAATGAAAAAAA<br>TGATCTATTGCAGATATTTGATGTAGTTTTCTTTTTTAAATTAA<br>ATCAGAAAGCTTGCGGCCGCACTCGAGTAACTAGGAAACCC<br>CTTGGGGCCTCTAATCGGGTCTTGAAGG | Amyloid precursor-like protein-2 (98%) |
| ATTCAGCGCTTGAATTCTCCTTTTCCGTTCCCAAGACATGTGC<br>AGCTCATCATCTGGCCATTTTCTCCCTGACGGTCCCACTTCTC<br>TCCAATCTTGTAGTTCACACCATTGTCATGGCACCATCTAGAT<br>GAATCACATCTGAAATGACCACTTCCAAAGCCTAAGCACTGG<br>CACAACAGTTTAAAGCCTGATTCAGACATTCGTTCCCACTCA<br>TCTCCAACGGCATAATGGGAAACTGTGTAGGGGTCAAAGCA<br>CGAGTCATCCGTAGGTTGGTTCAAGCCTTCGTTGACAGAGTT<br>GCCCACGGTAACAACCTCTTCCCGAAGCTTGCGGCCGCACTC<br>GAGTAACTAGTTAACCCCTTGGGGCCTCTAAACGGGTCTTGA<br>GGGGTTAAC | Fibronectin (90%) |
| CAGGAGAATGGTGTGAACCGGGGAGGCGAAGCTTGCAGTGA<br>GCCAAGATCGTGCCACTGCACTCCAGCCTGGGCGACAGAGC<br>GAGACTCCATCTCAAAAAAAAAGAAAAATAAATAAATAAATA<br>AATAAATTATCCTTCCACTATCTGCCACCTGAGGTAAGAGCAG<br>CCAGGGGATGCCATTTATCAACCCTATTTTTGCCACATAGCAG<br>TCTTACCAGGAATTGCTTTGCTTTATGTCTACTTGTCATTAAA<br>ACGTAGAGTTATACAACCTTGCAGATAAGCTTGCGGCCGCAC<br>TCGAGTAACTAGTTAACCCCTTGGGGCCTCTAAACGGGTCTT | No identification |

| | |
|---|---|
| GAGGGGTTAAC | |
| CATTTTTATAACCTGGCAAAATCTTGTTAATGTCATTGCTAAA<br>AAATAAATAAAAGCTAGATACTGGAAACCTAACTGCAATGTG<br>GATGTTTTACCCACATGACTTATTATGCATAAAGCCAAATTTC<br>CAGTTTAAGTAATTGCCTACAATAAAAAGAAATTTTGCCTGCC<br>ATTTTCAGAATCATCTTTTGAAGCTTTCTGTTGATGTTAACTG<br>AGCTACTAGAGATATTCTTATTTCACTAAATGTAAAATTTGGA<br>GTAAATATATATGTCAATATTTAGTAAAGCTTGCGGCCGCACT<br>CGAGTAACTAGTTAACCCCTTGGGGCCTCTAAACGGGTCTTG<br>AGGGGTTAAC | No identification |
| AAAAGTCCACACTTTAAGTTTTTCCCCACCTCCACTTTTTAAC<br>TTCTTATTTTTTCTCTTTATGTCTTATTGCAATGTTTCTTGAAA<br>AATCAGTTATTATTTTAATTGTTTCATTATTTAGTCTTTCTACGT<br>AAGAGTGTTTTACACAGCAAAATTACAGTGTTATAGTATTATG<br>TGTTTTACCCTGTGCTATTACCAGTGAGTTTTGTACCTTCAGA<br>TGATTTTTTATTGATCATGAACATGCTTTTTTTCAGATTGCAGA<br>ACTTCCTTTAGCATTTTTTATAGGACTGGTCTAGTGTTGATGA<br>AATCCCTCAGCTTTCCTTTGTCTGGAAAAGTTTTTGTTTCTCT<br>TTCATGGTTGAATAAGGCTTTTGCTGGACATACTATTCTAGGA<br>TAAAAGTTTTTTTTCTTCATCACTTAAATAAGTCATACCACTCT<br>CACCTCTCCTGTAAAGTTTCCACTGAAATGTCTGCTGCCAGA<br>TGTATTGGAACTCCATTGTATGTTATTAGGTTTTATTTTTTTTCT<br>CTTGCTGCTTTTAGGACTTTTTTTAAACCTTTGACATTTGGAA<br>GTTTGATTATTAAATGCTTTGAGGTATTTCTTTTTGGGTTGTGT<br>TCTATTACTTTCTTGTACTTAAATATGGATGTCTTTCTCTAGGT<br>TTGGGAATTTATCTGATATTATCCTTTTGAATAAGCTTGCGGCC<br>GCACTCGAGTAACTAGTTAACCCCTTGGGGCCTCTAAACGGG<br>TCTTGAGGGGTTAACTAGTTACTCGAGTGCGGCCGCAAGCTT<br>ATTCAATAGGTTAATATCAGATAATTTATAAATCCAGAGAAAG<br>ACATACATATTTAAGTACAAGAAAGTAATAGAACTCAACCCA<br>CCCAGAAATAACTTCCCTGAATCTATTAATATTCATCTCAAATG<br>GCAAGGGTTAAAAAAGTCCTCAAAGCGCCAGAGGAAAAAA<br>ATAAACCCATAACTTTATTGAG | No identification |
